# Supplementary material for: Biosynthesis of Smaller-Sized Platinum Nanoparticles Using the Leaf Extract of Combretum erythrophyllum and Its Antibacterial Activities
Source: Antibiotics (Basel). 2021 Oct 20;10(11):1275. doi: 10.3390/antibiotics10111275 (PMC8614812; doi:10.3390/antibiotics10111275)
Supplement: Supplementary file 1 [file antibiotics-10-01275-s001.zip › antibiotics-1409546-supplementary.pdf]

Supplementary Materials

Biosynthesis of Smaller-Sized Platinum Nanoparticles Using the Leaf Extract of *Combretum erythrophyllum* and Its Antibacterial Activities

Olufunto T. Fanoro, Sundararajan Parani, Rodney Maluleke, Thabang C. Lebepe, Rajendran J. Varghese, Nande Mgedle, Vuyo Mavumengwana and Oluwatobi S. Oluwafemi

Table S1. Antibacterial Study of PtNPs, CE Extract and Streptomycin via Minimum Inhibitory Concentration Method.

S.N.	Bacterial Strain	Gram +/–	PtNPs (µg/mL)	CE Extract (µg/mL)	Streptomycin (µg/mL)
1	*Staphylococcus epidermidis* (Se) (ATCC14990)	+Ve	3.125	-	1.56
2	*Proteus mirabilis* (Pm) (ATCC 7002)	−Ve	3.125	-	1.56
3	*Escherichia coli* (Ec) (ATCC 25922)	−Ve	-	-	1.56
4	*Staphylococcus aureus* (Sa) (ATCC 25923)	+Ve	-	-	1.56
5	*Klebsiella pneumoniae* (Kp) (ATCC 13822)	−Ve	2000	125	1.56
6	*Klebsiella oxytoca* (Ko) (ATCC 8724)	−Ve	1.56	-	1.56
7	*Mycobacterium smegmatis* (Ms) (MC 2155)	+Ve	-	2000	1.56
8	*Klebsiella aerogenes* (Ka) (ATCC 27853)	−Ve	1.56	-	1.56
9	*Bacillus cereus* (Bc) *(ATCC 10876)*	+Ve	1.56	-	-
10	*Proteus vulgaris* (Pv) (ATCC 6380)	−Ve	-	-	-
11	*Bacillus subtilis* (Bs) (ATCC 19659)	+Ve	500	2000	-
12	*Enterococcus faecalis* (Ef) (ATCC 13047)	+Ve	2000	-	-
